# Impact of Intermittent Hypoxia on Sepsis Outcomes in a Murine Model

**DOI:** 10.1038/s41598-019-49381-w

**Published:** 2019-09-09

**Authors:** Kun-Ta Chou, Shih-Chin Cheng, Shiang-Fen Huang, Diahn-Warng Perng, Shi-Chuan Chang, Yuh-Min Chen, Han-Shui Hsu, Shih-Chieh Hung

**Affiliations:** 1 Institute of Clinical Medicine, National Yang-Ming University, Taipei, Taiwan, ROC; 2 Faculty of Medicine, School of Medicine, National Yang-Ming University, Taipei, Taiwan, ROC; 3 Institute of Emergency and Critical Care Medicine, National Yang-Ming University, Taipei, Taiwan, ROC; 4 Center of Sleep Medicine, Taipei Veterans General Hospital, Taipei, Taiwan, ROC; 5 Department of Chest Medicine, Taipei Veterans General Hospital, Taipei, Taiwan, ROC; 6 Divsion of Infection, Department of Medicine, Taipei Veterans General Hospital, Taipei, Taiwan, ROC; 7 Division of Thoracic Surgery, Department of Surgery, Taipei Veterans General Hospital, Taipei, Taiwan, ROC; 8 Institute of Molecular Medicine, National Tsing Hua University, Hsinchu, Taiwan, ROC; 9 Department of Medical Science, National Tsing Hua University, Hsinchu, Taiwan, ROC; 10 Institute of Biomedical Sciences, Academia Sinica, Taipei, Taiwan, ROC; 11 Department of Orthopaedics, and Integrative Stem Cell Center, China Medical University Hospital, Taichung, Taiwan, ROC; 12 Institute of New Drug Development, Biomedical Sciences, China Medical University, Taichung, Taiwan, ROC

**Keywords:** Obesity, Respiratory tract diseases, Experimental models of disease

## Abstract

Sleep apnea has been associated with a variety of diseases, but its impact on sepsis outcome remains unclear. This study investigated the effect of intermittent hypoxia [IH]–the principal feature of sleep apnea–on murine sepsis. 5-week-old male C57BL6 mice were assigned to groups receiving severe IH (O2 fluctuating from room air to an O2 nadir of 5.7% with a cycle length of 90 seconds), mild IH (room air to 12%, 4 minutes/cycle), or room air for 3 weeks. Sepsis was induced by cecal ligation and puncture and survival was monitored. Sepsis severity was evaluated by murine sepsis scores, blood bacterial load, plasma tumor necrosis factor-α [TNF-α]/interleukin-6 [IL-6] levels and histopathology of vital organs. Compared with normoxic controls, mice subjected to severe IH had earlier mortality, a lower leukocyte count, higher blood bacterial load, higher plasma TNF-α and IL-6 levels, more severe inflammatory changes in the lung, spleen and small intestine. Mice subjected to mild IH did not differ from normoxic controls, except a higher IL-6 level after sepsis induced. The adverse impact of severe IH was reversed following a 10-day normoxic recovery. In conclusion, severe IH, not mild IH, contributed to poorer outcomes in a murine sepsis model.

## Introduction

Obstructive sleep apnea (OSA) is characterized by frequent pauses in breathing during sleep due to pharyngeal airway collapse. It affects an estimated 26% of the adult population^1^ and has been well-known for its linkage to cardiovascular comorbidities, such as hypertension, coronary artery disease, heart failure and arrhythmia^2,3^. Intermittent hypoxemia (IH) and sleep fragmentation have been thought to be the main mechanisms underlying this association^4^. The same insults are likely to affect other parts of the body outside the cardiovascular system^5^. In recent years, the association between OSA and other non-cardiovascular diseases/conditions has been explored, including cancer^6^, diabetes^7^, and neurologic diseases^8^.

Given that sleep plays a role in the maintenance of intact immune functions, disrupted sleep in OSA patients may have an adverse effect on immunity. We previously reported that OSA patients have a higher risk of pneumonia^9^ and poorer outcomes when confronting sepsis^10^. However, results from human studies were apt to be confounded by other factors coexisting in these patients, such as obesity, comorbid diseases or variable infection types/severities. In this study, we aimed to investigate whether IH–the principal component of OSA contributes to poorer outcomes in a murine sepsis model

## Materials and Methods

### Animals

**Figure 1:**
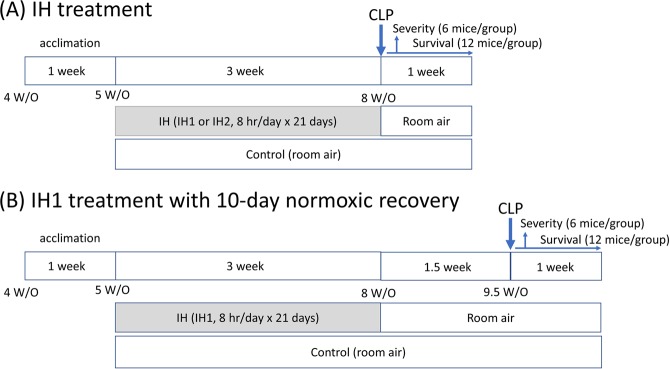
The study design.

This research was conducted according to the National Research Council guidelines and was approved by the Animal Care and Use Committee of Taipei Veterans General Hospital, Taipei, Taiwan (IACUC #2015-275). 4-week-old male C57BL/6 mice were purchased from the National Laboratory Animal Center (Taipei, Taiwan) and housed in a plastic chamber (Allentown, Xj cage, 7.63 in × 15.01 in × 5.13 in) at the Animal Center of Taipei Veterans General Hospital. Mice had ad libitum access to regular chow and water and were maintained under a 12-hour day/night cycle. Humidity was maintained at 63–72% and temperature was kept at 22–24 °C. After a 1-week acclimation to the environment, the mice (at the age of 5 weeks) were subjected to IH or room air (~21% O2) for 3 weeks. Sepsis was induced and the mice were monitored 1 week for survival. In separate experiments, mice were sacrificed 24 hours after CLP. The study flow is illustrated in the Fig. 1A. If survival differences were observed between specific groups in the initial CLP experiments, further experiments were arranged. We allowed IH-treated mice to recover in room air for an additional 10 days (normoxic recovery) and then CLP was implemented. Survival and sepsis scores were evaluated after surgery. (Please refer to Fig. 1B).

### Intermittent hypoxia

Cyclic fluctuation of the oxygen concentration in the chamber (FcO2) can be achieved by infusing nitrogen gas (N2) and oxygen gas (O2) alternatively through timed solenoid valves^11,12^ (E18107-151, Model: OC-04, HCS Co., TW). Gas flows were adjusted to achieve two IH protocols: severe IH (IH1) and mild IH (IH2). In the IH1 protocol, FcO2 fluctuated from room air to an O_2_ nadir of 5.7–6% with a cycle length of 90 seconds^12^. In the IH2 protocol, FcO_2_ cycled every 4 minutes from room air to 12%^13^.

FcO_2_ was monitored by an oxygen analyzer (MX300-I, Teledyne Analytical Instruments). IH was applied 8 hours per day (from 9 am to 5 pm) for 21 days in the IH (IH1 & IH2) groups and room air was with the normoxic control group^14^.

### Cecal ligation and puncture (CLP) model of sepsis

After 3-week treatment with IH or normoxia, sepsis was induced by cecal ligation and puncture (CLP)-the most frequently used model of polymicrobial sepsis, as described in the literature^15,16^. Briefly, after midline laparotomy under anesthesia, 50% of the cecum below the ileocecal valve was ligated, followed by a single through and through puncture (19-gauge needle). A small amount of stool was squeezed from the puncture holes. The cecum was relocated into the abdominal cavity, followed by closure of the peritoneum and abdominal wall. After surgery, 1 ml of pre-warmed saline (0.9% saline) was given subcutaneously.

### Sepsis severity

Sepsis severity was evaluated, including leucocyte count, plasma interleukin-6 (IL-6), tumor necrosis factor-α (TNF-α) levels and bacterial load in blood and the Murine Sepsis Score 24 hours after CLP^17,18^. The designated time was based on prior preliminary tests. Blood was collected into EDTA tubes by cardiac puncture for the determination of leucocyte count by Sysmex XT-1800i (Sysmex, Hyogo, Japan) and IL-6 and TNF-αlevels using commercial ELISA kits (eBioscience 88-7064-88 & 88-7324-88). To determine the bacterial load, the collected blood was plated on sheep blood agar plates. After a 24-hour incubation at 37 °C, the number of colony forming units (CFUs) was counted.

### Histopathologic evaluation

Tissue samples of vital organs from 5–6 mice each group, including the lung, spleen, small intestines, heart, brain, and kidney were collected 24 hours after CLP and fixed in 10% formalin, embedded in paraffin and sectioned at a thickness of 4 mm. The slides were stained with H&E (hematoxylin-eosin, Muto Pure Chemicals Co., Ltd.) and scored three images per sample under a light microscope 40X/100X in a blinded fashion (by Chen Jun & Wu Nian-Ting).

Histological changes of lungs and guts were scored by the criteria described in the literature^19,20^. Briefly, the morphologic changes of lungs were evaluated based on the presence of exudates, hyperemia/congestion, neutrophil infiltration, alveolar hemorrhage/debris and cellular hyperplasia. Each item is scored on a range from 0 (nil) to 3 (severe). The sum of scores of different mice was averaged^19^. Intestinal injury was scored according to Chang and colleagues, assessing subepithelial gap of the villus, capillary blood congestion, epithelial separation from the lamina propria, exfoliation of epithelium and lamina propria digested/disintegrated (score, 0–5)^20^. Changes of the spleens were scored based on the size and evenness of white pulps as follows: 0: normal; 1: few, scattered atrophic white pulps, 2: moderate amount of scattered atrophic white pulps, 3: abundant atrophic white pulps with some normal white pulps interposed, 4: diffuse atrophic white pulps.

### Statistical analysis

Statistical analysis was conducted using IBM SPSS Statistics for Windows, Version 22.0 commercial software. (Released 2013. Armonk, NY: IBM Corp.) Comparison of parametric variables (including pre- & post-CLP leukocyte counts, IL-6 level, sepsis score, scores of histologic changes) between 3 groups was carried out by Analysis of variance (ANOVA) with Bonferroni method for post-hoc multiple comparisons whereas non-parametric variables (TNF-α, bacterial load) were analyzed using Kruskal–Wallis test with Dunn method for post-hoc pairwise comparison. Comparison between two groups (pre- vs post-CLP/IH1 vs control) was made by Student’s t test (leukocyte count, IL-6, sepsis scores) or Mann-Whitney U test (TNF-α) Survival analysis was conducted utilizing the Kaplan-Meier method, with statistical significance determined by the log-rank test. Statistical significance was inferred with *p* values less than 0.05. The figures were plotted using GraphPad Prism 7.0 (GraphPad Software, Inc., San Diego, CA).

## Results

### Intermittent hypoxia

**Figure 2:**
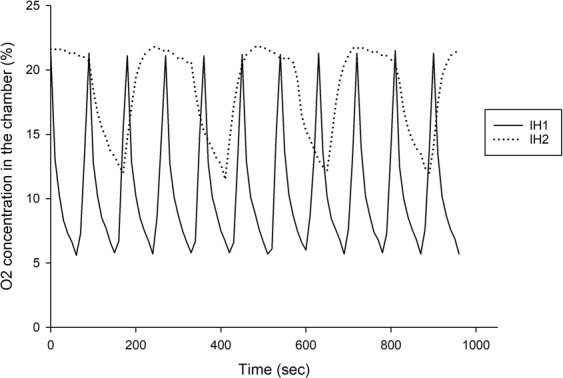
Profile of Intermittent hypoxia of the IH1 and IH2 protocols.

Figure 2 revealed FcO2 profiles in the IH1 and IH2 protocols. IH1 mimics severe sleep apnea with a rate of 40 apneas per hour whereas IH2 mimics milder disease with rate of 15 apneas per hour.

### Effect of IH and CLP

**Figure 3:**
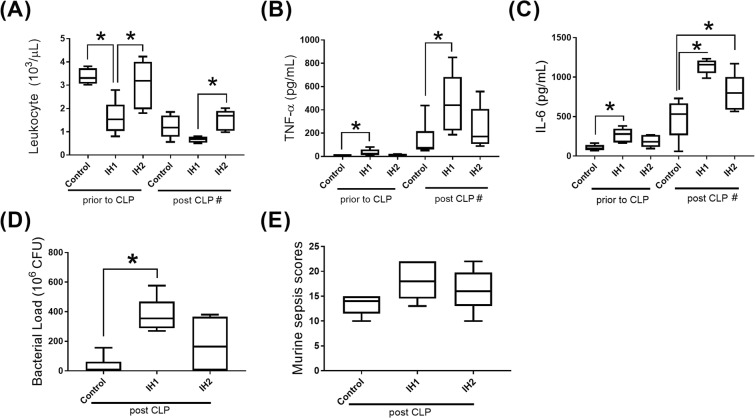
Levels of Inflammatory biomarkers and sepsis severity in mice. (**A**) leukocyte count, (**B**) TNF-α, (**C**) IL-6 (**D**)bacterial load, (**E**) sepsis scores. Post-CLP IH2 and control groups: n = 6; other groups: n = 5. *p < 0.05; ^#^p < 0.05 for comparison between before and after CLP in each of the three groups (IH1/IH2/control).

Prior to CLP, IH1-treated mice had a lower leucocyte count, higher plasma IL-6 and TNF-α levels compared to controls (p < 0.05, Fig. 3A–C). Though IH1-treated mice also had a lower leucocyte count than IH2-treated mice, the two groups did not differ in IL-6 and TNF-α levels.

After induction of sepsis with CLP, the IH1 group still had higher IL-6 and TNF-α levels than controls, but the difference in the leukocyte count between the two groups did not reach statistical significance. There was no difference between the IH2 group and controls, except a higher IL-6 level in the IH2 group.

No matter which type of intervention was applied (IH1/IH2/control), the effect of CLP was consistent, contributing to a decline in the leukocyte count and an increase inIL-6 and TNF-α levels in each of the three groups (IH1/IH2/control, Fig. 3A–C).

### Sepsis severity and mortality

**Figure 4:**
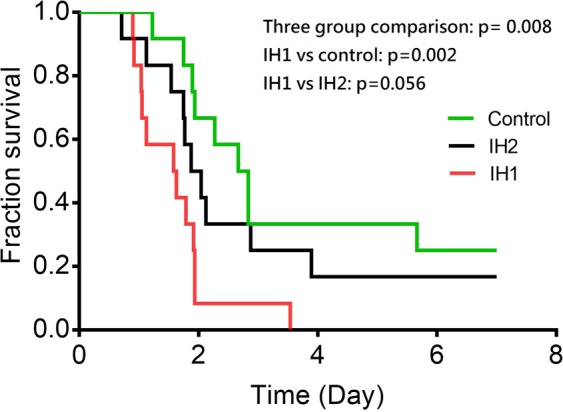
Survival after cecal ligation and puncture. In comparison to normoxic controls, the IH1 group had significantly earlier mortality. The difference between IH1 and IH2 showed a trend toward statistical significance. p = 0.008 for three-group comparison. IH1 vs control: p = 0.002, IH1 vs IIH2: p = 0.056. n = 12 for each group.

After sepsis induced by CLP, mice in the IH1 group had higher bacterial load (Fig. 3D) than normoxic controls. The sepsis scores did not differ between groups (Fig. 3E). The IH1 group had a significantly earlier mortality than controls (Fig. 4)

### Histopathology

**Figure 5:**
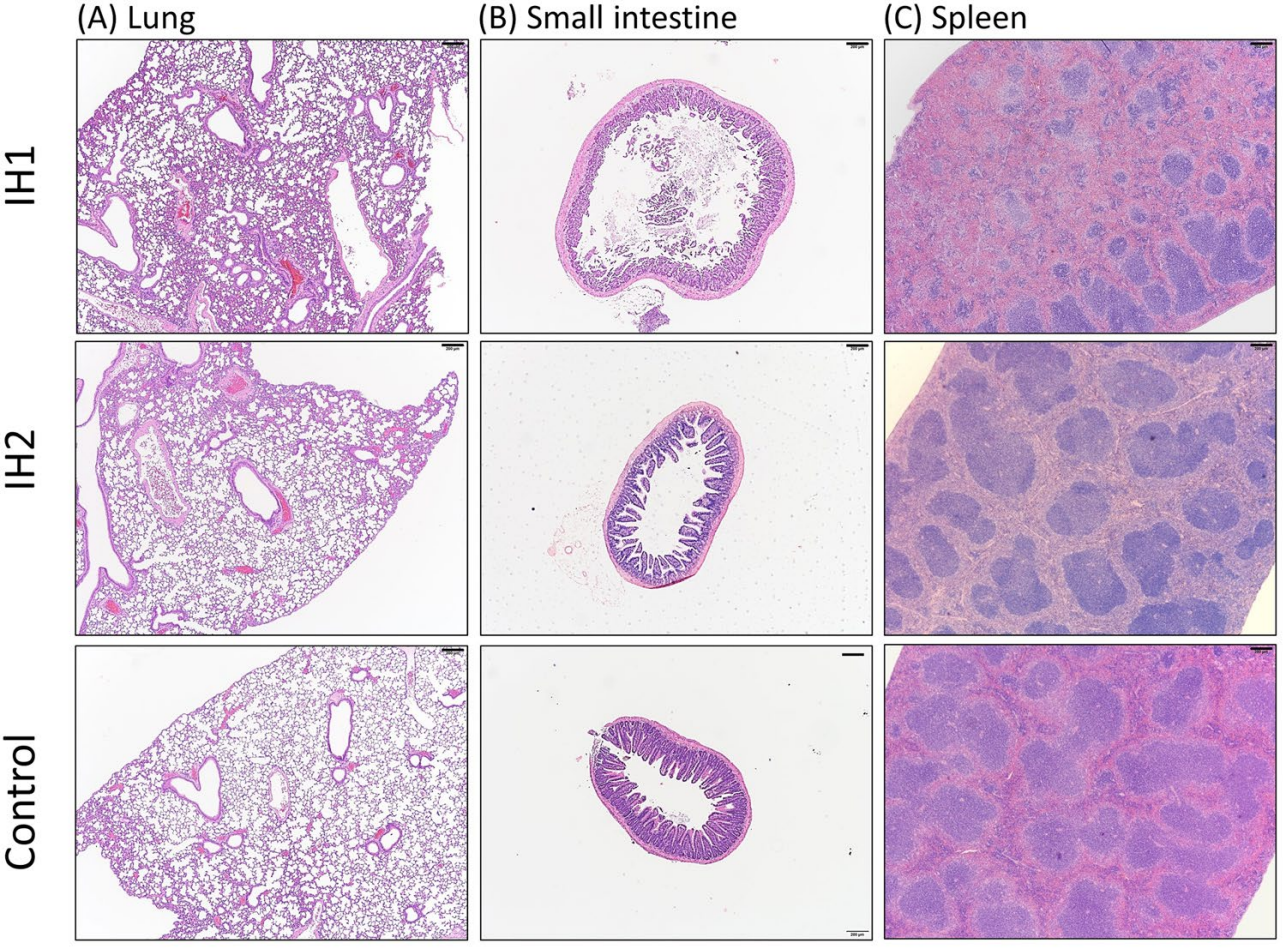
Histopathologic evaluation with hematoxylin-eosin staining (magnification 40×). showed more prominent inflammatory changes in the lung, small intestine and spleen in the IH1 group than in the IH2 group and normoxic controls. Bar: 200 μm.

**Figure 6:**
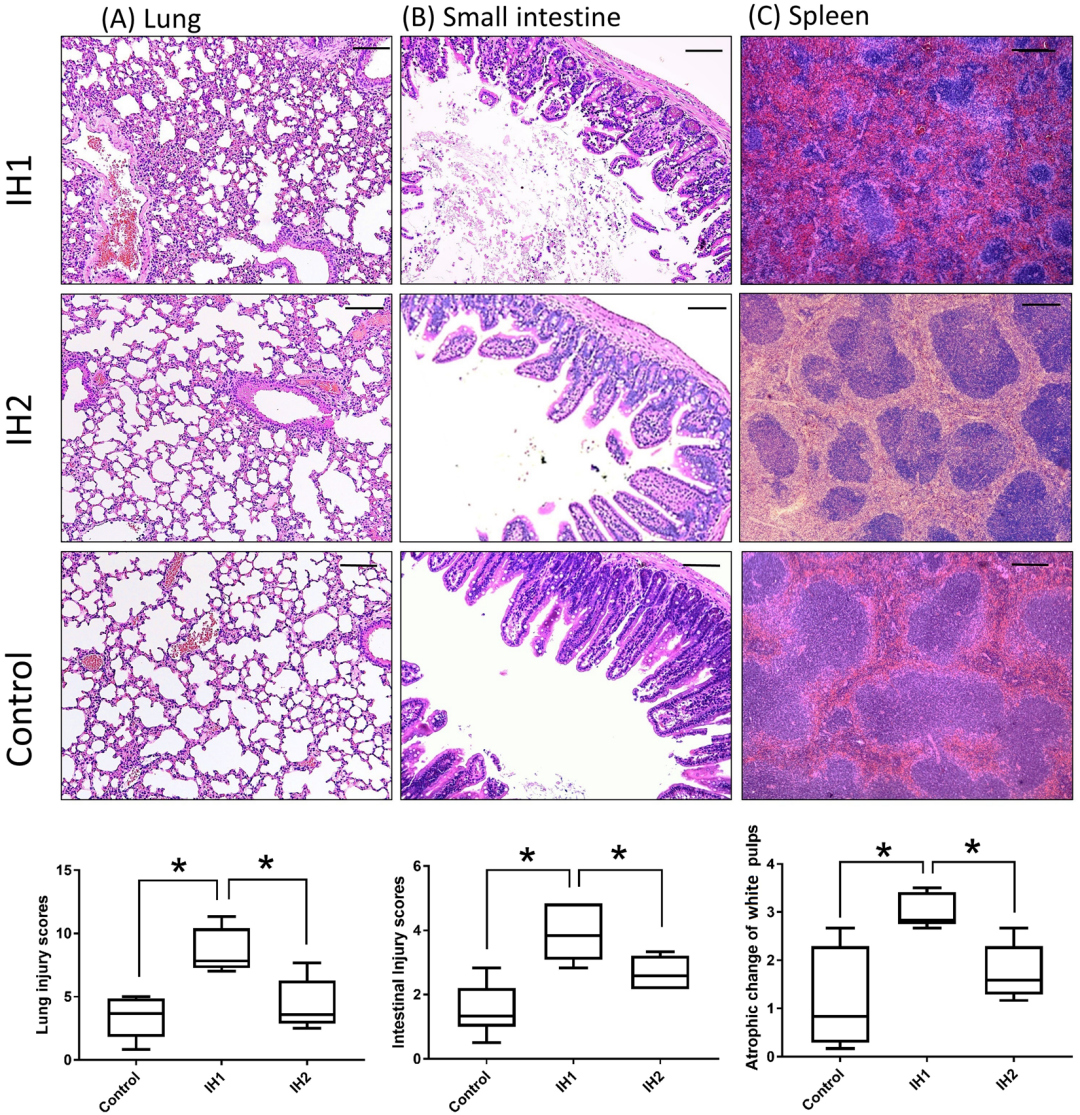
Histopathologic evaluation with hematoxylin-eosin staining (magnification 100×). The IH1 group had more severe inflammatory change than the IH group or normoxic controls. Scores of organ damage were shown on the last row. Bar: 100 μm.

Histopathologic examination showed inflammatory changes in the lung, spleen and small intestine were more prominent in the IH1 group than in the IH2 or the control group (Fig. 5 and 6) while chagnes of the other organs did not differ between groups (not shown). Lung specimens from the IH1 group showed more severe exudative changes, thickened alveolar interstitium and heavier infiltration of inflammatory cells into the intra-alveolar and interstitial spaces compared to the IH2 or controls (Fig. 6A). The intestines of the mice in the IH1 group had more widespread epithelium destruction and inflammatory cell infiltration than the other two groups (Fig. 6B). As for the spleen, shrinkage of white pulps was more severe in the IH1 group than the other two groups (Fig. 6C).

### Normoxic recovery

**Figure 7:**
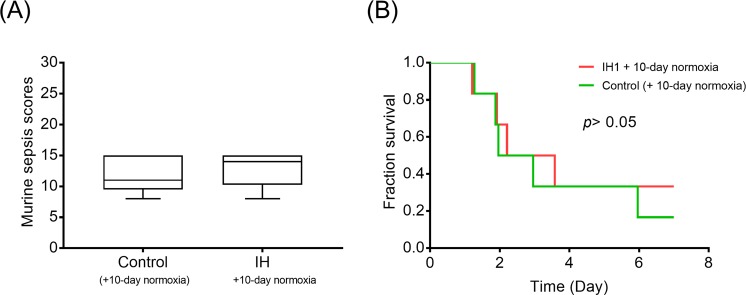
Normoxic recovery. With a 10-day normoxic recovery following the IH1 protocol, sepsis severity (**A**) and mortality (**B**) did not differ significantly between the IH1 group and normoxic controls. n = 6 for each group.

Given that a survival difference was evident only between the IH1 and the control group, we rendered IH1-treated mice to recover in a normoxic condition for 10 days following IH treatment. The results showed that sepsis severity and mortality of the two groups did not differ after a 10-day normoxic recovery (Fig. 7).

## Discussion

In this study, we found that a 3-week treatment with severe IH prior to CLP contributed to increased severity of murine sepsis and earlier mortality. The results support our previous human research, which revealed patients with OSA had a higher risk of mortality when dealing with sepsis^10^.

Research has revealed immunologic derangement in patients with OSA, such as elevated inflammatory cytokines/mediators in blood and abnormally-functioning immune cells^21–23^. In particular, Cubillos-Zapata *et al*. reported that PD-L1/PD-1 crosstalk was upregulated in OSA patients, which may decrease CD8+ T-cell activation and cytotoxic activity^21^. Another study by Said *et al*. showed a decreased phagocytotic capacity of neutrophils in OSA patients^22^. Therefore, it seems reasonable to postulate that OSA is likely to facilitate the occurrence of infection or to worsen infection-associated outcomes.

However, research examining whether OSA patients are prone to infection or poorer outcome in a clinical setting is limited. Some studies have shown sleep apnea patients are more susceptible to pneumonia^9,24–26^ and are likely to have a poorer outcome related to infection^10,27^ although other studies reported neutral or contradictory findings^28,29^. Nevertheless, the available information from these studies should be interpreted cautiously due to the confounding conditions that commonly coexist with OSA. For instance, most OSA patients are obese and obesity may predispose subjects to infection or significantly impact infection-related outcomes^30,31^. A similar caution should apply to the age of patients^32^ and comorbidities associated with OSA (for example, diabetes^33,34^ and stroke^35^). Hence, we conducted this animal study to highlight the impact of IH on sepsis and to avoid interference from the confounders.

In this study, even before CLP, mice subjected to severe IH (IH1 protocol) had higher plasma IL-6 and TNF-α levels and a lower leucocyte count. The elevation of inflammatory cytokines is compatible with the study by He *et al*.^36^, showing that Wistar rats subjected to a 8-week treatment of severe IH (with an O2 nadir to 5%) had the highest IL-6 and TNF-α levels in blood compared to rats treated with milder IH (an O2 nadir to 7.5%/10%) or normoxia. Similarly, in human OSA, elevation of plama IL-6 and TNF-α was observed following the first episode of apnea causing an SaO2 < 85%^23^ and can persist long as a part of systemic inflammation^4,37^. With respect to the lower leukocyte count after severe IH in this study, literature regarding the impact of IH on the leukocyte count in the peripheral blood of mice is limited. However, Alvarez-Martins *et al*. showed Wistar rats subjected to a 32-day IH treatment, compared to normoxia, had a lower circulating leukocyte count despite the p value not reaching statistical significance^38^. Since the above-mentioned changes can also occur in murine sepsis alone^15,39,40^, it is probable to be augemted when IH treatment is followed by sepsis.

Moreover, our results showed that severe IH (IH1 protocol), not mild IH (IH2 protocol) significantly contributed to more severe murine sepsis outcomes. Despite the possibility that the sample number in each group was too small to highlight the impact of the IH2 protocol, the adverse impact of severe OSA is indeed more prominent than mild OSA based on the results of clinical research^41,42^. Severe OSA is more closely linked to adverse health consequences, including cardiovascular, cardiometabolic, and psychological comorbidities and overall mortality, but its association with mild disease remains unclear^41,42^. Although our conclusion may be limited for severe IH (IH1), the differential impact of severe and mild IH (IH2) is compatible with the clinical findings^41,42^.

In addition to this study eliminating the confounding of obesity as seen in OSA patients, another strength is the inclusion of mice undergoing normoxic recovery after IH1, which mimics the condition in OSA patients under effective treatment, such as continuous positive airway pressure (CPAP) therapy, which is able to keep the airway patent and prevent desaturation. The reversal of severe IH’s impact through normoxic recovery further strengthens our conclusion.

However, it is not always possible to recover from the insults of IH produced by OSA in humans. Take the most common symptom-excessive daytime sleepiness as an example. 6–14% of patients under adequate CPAP treatment were still bothered by residual sleepiness^43,44^. This can be, at least partly, attributed to irreversible cellular injury in brain regions that control sleep wake regulation^45–47^. The subtle injury in these patients have not yet ended in an overt neurologic disease, but still can present with sleepiness despite treatment, not to say those who established overt neurologic diseases, such as stroke. Concerning the impact of IH on immunity, the existing evidence is still limited. The current study is the first animal study to show the adverse impact of IH on sepsis outcomes, but further research is warranted to determine whether this is contributory in humans.

Despite the aforementioned strengths, the study still has some limitations. Firstly, the frequency and magnitude of hypoxia vary across OSA patients. The two IH protocols we adopted in this study still cannot capture the entire spectrum of IH with its varying severity. It also should be noted that IH is just one aspect of the pathophysiologic characteristics of OSA. Other features of OSA, such as sleep fragmentation, and exaggerated intrathoracic pressure changes were not included in our model^4^.

Secondly, different IH profiles may provoke divergent responses, depending on the characteristics of IH, such as severity/duration of hypoxia and cycle frequency^48^. Less frequent, milder hypoxic exposures or short-term exposure to IH may bring beneficial effects; this has been demonstrated in the field of sports and altitude training^49,50^. Whether the IH-provoked response is beneficial or harmful also depend on the ability of affected subjects to adapt themselves to IH insults; hence inter-individual biological variance in OSA patients must be taken into account^48^. Furthermore, the use of young animals in this study is also a limitation although we performed CLP on the mice at the age of 8 week per the suggestion of the article (7–9 weeks) by Toscano *et al*.^15^. However, young mice may have different responses to IH or infection from the aged mice. Lastly, we used the CLP model in this study to induce intra-abdominal sepsis in mice and to demonstrate the impact of IH. In real practice, infection in other sources, such as the lung and urinary tract, may also be responsible for sepsis. It is not known if the effect of IH is consistent when the origin of sepsis is other than the abdomen. Furthermore, tolerance to hypoxic insults may differ between species. Whether our conclusion can be extrapolated to the human condition warrants exploration in further research.

## Conclusions

We found that, in a murine sepsis model, a 3-week treatment of severe IH, not mild IH, contributed to increased severity and earlier mortality. This adverse effect was reversed by a 10-day normoxic recovery following severe IH.

## Data Availability

All data can be provided to the readers. Please contact with the corresponding authors Prof. Hung, Shih-Chieh or Prof. Cheng, Shih-Chin.
